# An Assessment of Subsurface Residual Stress Analysis in SLM Ti-6Al-4V

**DOI:** 10.3390/ma10040348

**Published:** 2017-03-27

**Authors:** Tatiana Mishurova, Sandra Cabeza, Katia Artzt, Jan Haubrich, Manuela Klaus, Christoph Genzel, Guillermo Requena, Giovanni Bruno

**Affiliations:** 1 Bundesanstalt für Materialforschung und-prüfung (BAM; Federal Institute for Materials Research and Testing), Unter den Eichen 87, 12205 Berlin, Germany; sandra.cabeza@bam.de (S.C.); giovanni.bruno@bam.de (G.B.); 2 Institute of Materials Research, German Aerospace Center (DLR; Deutsches Zentrum für Luft-und Raumfahrt), Linder Höhe, 51147 Cologne, Germany; katia.artzt@dlr.de (K.A.); jan.haubrich@dlr.de (J.H.); guillermo.requena@dlr.de (G.R.); 3 Helmholtz-Zentrum Berlin für Materialien und Energie GmbH, Hahn-Meitner Platz 1, 14109 Berlin, Germany; klaus@helmholtz-berlin.de (M.K.); genzel@helmholtz-berlin.de (C.G.); 4 Institute of Physics and Astronomy, University of Potsdam, Karl-Liebknecht-Straße 24/25, 14476 Potsdam, Germany

**Keywords:** selective laser melting, additive manufacturing, heat treatment, Ti-6Al-4V, synchrotron X-ray diffraction, residual stress

## Abstract

Ti-6Al-4V bridges were additively fabricated by selective laser melting (SLM) under different scanning speed conditions, to compare the effect of process energy density on the residual stress state. Subsurface lattice strain characterization was conducted by means of synchrotron diffraction in energy dispersive mode. High tensile strain gradients were found at the frontal surface for samples in an as-built condition. The geometry of the samples promotes increasing strains towards the pillar of the bridges. We observed that the higher the laser energy density during fabrication, the lower the lattice strains. A relief of lattice strains takes place after heat treatment.

## 1. Introduction

Additive manufacturing (AM) is still a rather new technique for producing metallic components. As opposed to conventional subtractive manufacturing technologies like milling or drilling, AM allows the building up of components by means of wire-fed, powder-fed, or powder-layer processes. AM opens up new opportunities for manufacturing highly complex, load- and lightweight optimized near-net shape components, which are particularly interesting for small series of parts. Notably, the manufacturing costs and lead time in an AM scale, mainly with part mass, and are only weakly dependent on the component complexity, in contrast to conventional processing technologies [1]. Among the family of additive manufacturing technologies, selective laser melting (SLM) has gained wide acceptance. It is a powder-bed fusion technique that enables the production of very fine and hollow structures, such as inner cooling channels for turbine blades or high-speed cutting tools.

However, even today, AM technologies and materials are still far from mature, and hinder AM from being exploited at its full potential. In SLM, multiple process parameters (e.g., laser power, powder layer thickness, laser scanning velocity, and scan strategy), as well as the rheological powder properties, influence the resulting material properties, i.e., porosity, cracks, surface properties, and microstructure, and hence, the mechanical properties. Additionally, high residual stresses are known to develop during the building process [2], affecting the mechanical performance, and possibly leading to undesired shape distortion, detachment from support structures, and even crack formation.

One particularly well-studied material for SLM is the α + β titanium alloy Ti-6Al-4V. The alloy has been developed to satisfy the needs of the aerospace industry, and it is the most widely used titanium alloy. This industry sector has gained more than six decades of experience with this material. As a consequence, the aerospace industry aims at accelerating the development and certification of the AM process by exploiting its expertise on this very alloy [3]. However, the mechanical performance of AM Ti-6Al-4V differs from that of forged or cast counterparts, owing to the different thermo-mechanical history introduced during manufacturing [4]. This necessitates the development of a new expertise for AM materials and their post-treatment possibilities.

Several works focus on the evolution of the microstructure during and after SLM production, and its connection to mechanical behaviour [5,6,7,8,9], generally showing that the Ti-6Al-4V as-built microstructure consists of brittle α′-martensite (hexagonal lattice) within prior-β grains, with a martensitic size significantly depending on the process parameters [5]. Phase transformations can be induced by heat treatments leading to a more ductile microstructure consisting of stable α (hcp) and β (bcc) phases. Commonly, the heat treatment is conducted after the building process (“extrinsic” or “ex-situ” after the AM process) (e.g., [10,11,12,13]), but approaches of in-situ heat treatment by preheating the baseplate or build space also exist [14,15].

Besides defects and the microstructure, the residual stresses in AM materials are critical for their mechanical performance and for retaining the ability to achieve near-net shape geometries (see e.g., [8,16,17,18]). High cooling rates at the order of 10^3^ K/s to 10^8^ K/s occurring in the SLM process [13,19,20,21], lead to significant residual stresses that can result in the distortion of samples and parts. Previous studies show that thermal residual stresses in SLM parts are primarily caused by two mechanisms: the temperature gradient, and the cool-down phase of the molten top layer [2]. Additionally, the influence of the number of layers, the thickness of the layers, the geometry, and the scanning strategy for Ti-6Al-4V parts have been investigated [2,16]. The results of the cited works show an increase of residual stress for a large number of layers (i.e., larger sample height), while the preheating of the base plate during scanning decreases the residual stress. Finite element thermo-mechanical models of residual stress have been reported in [22], showing the dependence of the residual stress state on the scanning strategy. Typically, the highest residual stresses were reported along the scanning direction. Besides the adjustment of the SLM scan strategies [16], the most common and versatile approach to reduce residual stresses of AM parts, prior to separation from the baseplate, is a “stress relief heat treatment” (e.g., 650 °C for 3 h) [8,9,23].

In most of the cases, residual stresses were determined by destructive methods (contour method [24], hole drilling) or laboratory X-ray diffraction [2,25]. In particular, the latter only presents information on the outer surface layers (i.e., a few tens of microns).

In the present work, we investigated the development of residual stresses in SLM-fabricated bridge specimens as a function of the laser process energy density introduced during manufacturing, in order to identify suitable SLM processing parameter limits. To fulfil this aim, Ti-6Al-4V samples in the form of bridges were used also to assess the parameters influencing the residual stress state and to guide the development of SLM processes [16,26]. We first studied the curvature of bridges, which occurred after their removal from the baseplates (different bridges were produced by varying the laser scan speed). Subsequently, two bridges built at two selected conditions in the processing window were investigated by synchrotron energy-dispersive diffraction, to study the residual stress state in the subsurface region in further detail. Energy-dispersive diffraction performed in reflection mode using high energy synchrotron radiation allowed us to sample the materials near the surface region and to record complete diffraction patterns under fixed (but arbitrary) scattering angles [27]. Furthermore, the use of synchrotron radiation allows the use of small gauge volumes, and thereby enables high spatial resolution in the subsurface region.

## 2. Materials and Methods 

**Figure 1:**
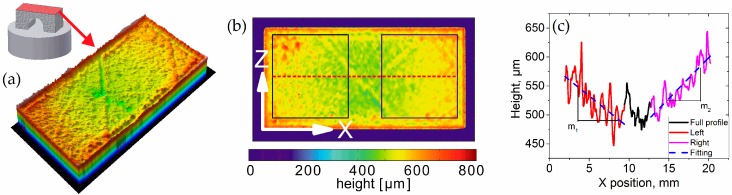
Determination of the deflection angle α. For the part of the profile inside the selected areas on the left and on the right side, the slopes *m*_1_ and *m*_2_ are calculated with respect the longitudinal direction. The slopes are converted to deflection angles (here: α_1_ = −0.63° and α_2_ = +0.81°). (**a**) Top surface LSM; (**b**) selected area LSM; (**c**) height profile.

Bridge-shaped specimens were produced under an argon atmosphere using a SLM Solutions 280HL machine with a single IPG fibre laser (maximum power 400 W). The baseplate was heated to a fixed temperature of 200 °C during the SLM process. Plasma atomized (AP&C) Ti-6Al-4V ELI grade 23 powder with a particle size of *d*_90_ < 50 μm, as determined by laser diffraction (Beckman Coulter GmbH), was used for the SLM process. The bridge specimens had a length of 20 mm, a height of 8 mm, and a thickness of 10 mm (Figure 1a) [16].

Two sets of bridge-samples were produced using different scanning velocities at a constant laser power for the bulk processing parameters (Table 1), to systematically vary the energy density introduced by SLM. For all samples, a set of two contours and one intermediate line were initially produced, before the sample bulk was processed (with specific parameters). The contour parameters (100 W and 525 mm/s scan velocity) were fixed for this first study. For all bridges, a constant layer thickness of 30 μm and a chess-pattern scan strategy with a minimum field size of 5 mm were chosen. All bridges were built directly on the baseplate. Support structures were only used in the centre of the bridge arcs. After SLM fabrication, selected samples were subjected to a heat treatment at a temperature of 650 °C for 3 h under vacuum conditions, to relieve residual stresses. The porosity of sister samples, produced with the same scanning parameters, has been reported in [28]. The porosity volume fractions are reported in Table 1.

The residual stress state of the samples was characterized for three conditions: (1) as-built on the baseplate (BP); (2) after stress relief heat treatment on the baseplate (TT); and (3)directly released (wire-cut) from the baseplate (R) without TT. Measurements were performed on the same sample before (BP) and after cutting from the baseplate (R), while the heat treatment was performed on a sister sample.

The “as-built” surface roughness on the side surfaces of the SLM bridge samples was determined with a confocal ZEISS LSM 700 laser scanning microscope (Carl Zeiss AG, Oberkochen, Germany). Height maps from lateral surfaces were created for small areas of 1.2 × 1.2 mm^2^ (pixel size = 1.25 × 1.25 μm^2^ for each optical slice, 1.46 μm in height). The surface roughness parameters *S*_a_ (arithmetic mean height) and *S*_q_ (root mean square height) were calculated according to ISO 25178, using the analysis software Confomap^®^ (Carl Zeiss AG, Oberkochen, Germany).

The distortion angles of the bridge-shaped specimens [16] were measured at their top surfaces with the ZEISS LSM 700 confocal microscope (Figure 1). Incidentally, this can be used for a fast assessment of the trends of residual stress state in SLM samples. For the released specimens (R), the bottom facets were also measured. Distortion angles were computed by averaging the angles measured in two rectangular regions towards the edges of the bridges along the longitudinal axis (Figure 1b). Each angle was calculated from the slopes *m*_1_ and *m*_2_, respectively (Figure 1c). This procedure was repeated for each line parallel to the *X* axis, yielding the deflection angle as a function of the *Z*-coordinate. From this, the average over around 800 lines was taken, and the mean deflection angle α of the bridge and the standard deviation were calculated.

**Figure 2:**
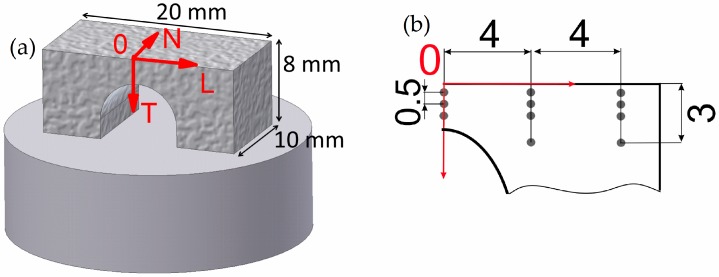
(**a**) Schematic representation of a bridge sample and its dimensions (T—transversal, L—longitudinal, N—normal); (**b**) specification of mapped area by synchrotron diffraction and specific points measured on the lateral surface.

Lattice strain measurements of selected samples were conducted at the synchrotron source BESSY II (HZB, Berlin) on the EDDI (Energy Dispersive Diffraction) beamline. This beamline provides a white beam with an energy range of about 10 keV–150 keV. The experiment was performed in a reflection set-up with a fixed diffraction angle of 2θ = 8°. The prismatic gauge volume is defined by the intersection of the incoming and diffracted beams. Primary slits with an opening of 500 × 500 μm² and secondary slits with a vertical opening of 30 μm were used for the determination of the transversal strain component. Measurements were carried out with the gauge volume fully immersed in the sample, but right at the surface. For the longitudinal strain component, the vertical opening of the primary slit was decreased to 350 μm, in order to decrease the length of the gauge volume owing to geometry limitations. Gauge volume lengths of 3.8 mm and 2.8 mm were obtained for the transversal and longitudinal strain components, respectively. For each measuring point, a ${\;\sin}^{2}\psi$ scan (see [29]) was performed with 10 ψ-tilts and an acquisition time of 30 s per tilt. The bridge-shaped specimen is depicted in Figure 2a, with a coordinate system being used during the measurements. The positions at which residual stresses were measured are shown as grey circles in Figure 2b.

**Figure 3:**
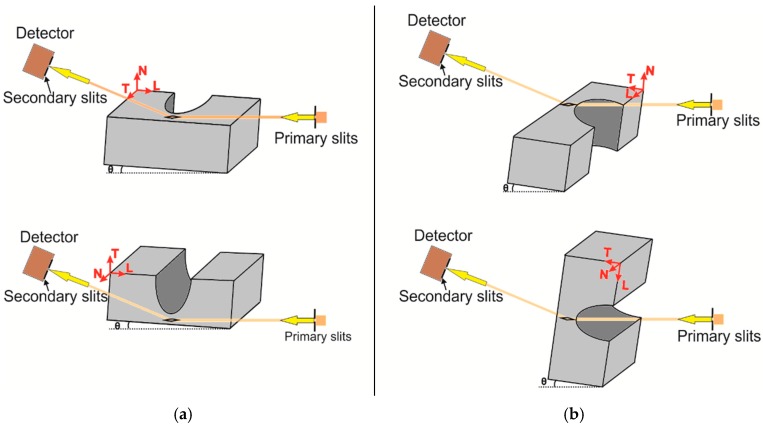
Sketch of residual stress measurement by sin^2^ ψ in reflection set-up for (**a**) transversal strain component; (**b**) longitudinal strain component (References system: L stands for longitudinal, T for transversal, N for normal components).

In the reflection set-up (near-surface residual stress state), the normal stress component is assumed to be negligible. Therefore, the absolute values of residual strain for the transversal (Figure 3a) and longitudinal (Figure 3b) components can be evaluated by considering the strain-free lattice parameters $d_{0}^{hkl}$, which were previously obtained from the measured $d_{\varphi \psi }^{hkl}-\sin^{2}\psi$ distributions in the strain-free $\psi^{*}$ direction (biaxial stress state): $\sin^{2}\psi^{*}=\frac{-2S_{1}^{hkl}}{\frac{1}{2}S_{2}^{hkl}}$. The $\sin^{2}\psi$ method yielded the residual stress in the azimuthal directions (here longitudinal and transversal), from the linear extrapolation of $d_{\varphi \psi }^{hkl}\;$ vs. ${\;\sin}^{2}\psi$ graphs. The longitudinal strain component could not be measured for the samples with a baseplate (BP and TT), due to the thickness of the baseplate.

The energy dispersive diffraction technique (the Bragg angle θ is kept fixed) allows us to obtain the signal from each crystallographic plane at a different energy:

$$d^{hkl}\left(Å\right)=\frac{6.199}{\sin\theta }\frac{1}{E^{hkl}\left(\mathrm{keV}\right)}$$

where $d^{hkl}$ is the lattice spacing for the crystallographic plane {*hkl*}, θ is the Bragg’s angle, and $E^{hkl}$ is the energy corresponding to the diffraction of the crystallographic plane {*hkl*}. It also allows probing different depths [30], whereby the penetration depth τ for the energy dispersive diffraction is determined by [27]:

$$\tau =\frac{\sin\theta }{2\mu \left(E\right)}\cos\psi$$

where θ is the Bragg’s angle, μ(E) is the linear X-ray absorption coefficient (energy dependent), and ψ is the angle of tilt.

For residual stress evaluation, we chose to analyse the non-overlapping diffraction lines of six crystallographic planes of α + α’ hexagonal Ti lattices.

The calculation of strains and stresses using the sin^2^ ψ method requires the elastic constants of the crystallographic plane under consideration. Diffraction elastic constants (DEC) of α were obtained according to the Eshelby/Kröner model [31,32] (Table 2). Since the reflections of α and α’ proved to be indistinguishable in the diffractograms, and no peak asymmetry was observed, only the α elastic constants were considered, to simplify the analysis. The small difference between the elastic constants of α and α’ does not affect the stress values above the error bar, and thereby the interpretation of the results. The diffraction peaks of the body-centred cubic β phase were not detectable, because of its low volume fraction [14,33]. The samples exhibited weak β—Ti peaks only after heat treatment, which were not usable for stress analysis. DEC $s_{1}$ and $s_{2}$ are connected with elastic constants by the following equations:

$$-s_{1}^{hkl}=\;\frac{\nu^{hkl}}{E^{hkl}}\text{ and }\frac{1}{2}s_{2}^{hkl}=\;\frac{1+\nu^{hkl}}{E^{hkl}}$$

where $\nu^{hkl}\;\mathrm{and}\;E^{hkl}$ are the plane-specific Poisson’s ratio and Young’s modulus, respectively.

## 3. Results

**Figure 4:**
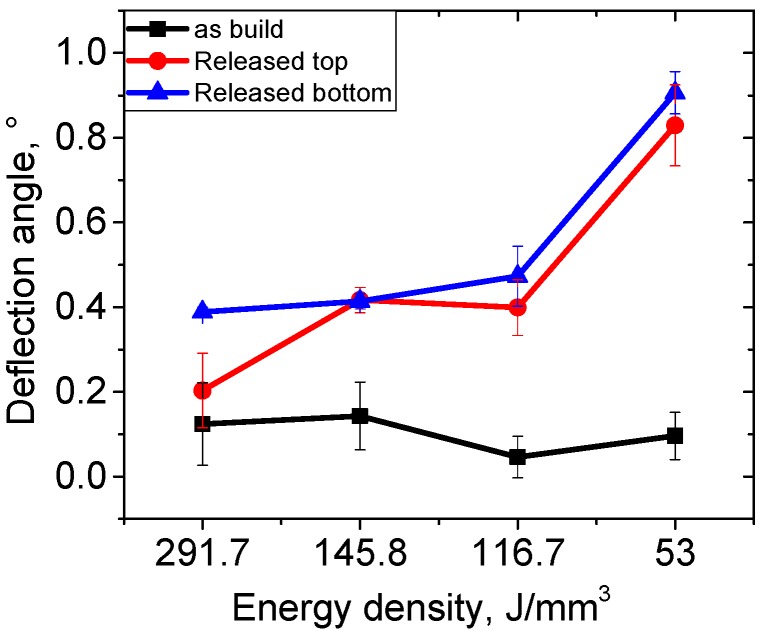
Distortion angles as a function of SLM process energy density (*E*_V_).

A first assessment of the residual stress in the SLM samples as a function of the process energy density (*E*_V_) is obtained from distortion angle measurements in the released bridge specimens. It discloses that the deformations strongly increase for *E*_V_ < 116 J/mm^3^, i.e., scanning velocities exceeding 500 mm/s (Figure 4). The deflection angle was measured at the top and bottom surfaces of the bridge specimens, after removing them from the baseplates by means of spark erosion. Apparently, for sufficiently high *E*_V_ (smaller laser scan velocities) such as those used for the samples A3 and A4, the distortion angles are similar. Prior to release from the baseplate (as-built condition), none of the samples showed any deformation within the accuracy of the measurement, and the obtained geometries retained their near-net shape. Using SLM process parameters close to the ones corresponding to *E*_V_ = 116.7 J/mm^3^, may provide a processing window with comparably low distortion angles (Figure 4), while at *E*_V_ much lower than ~53 J/mm^3^, highly distorted samples were obtained in a significantly different material state. Residual stress measurements were conducted for SLM parameters A4 (optimum window) and A10 (above the optimum window). 

**Figure 5:**
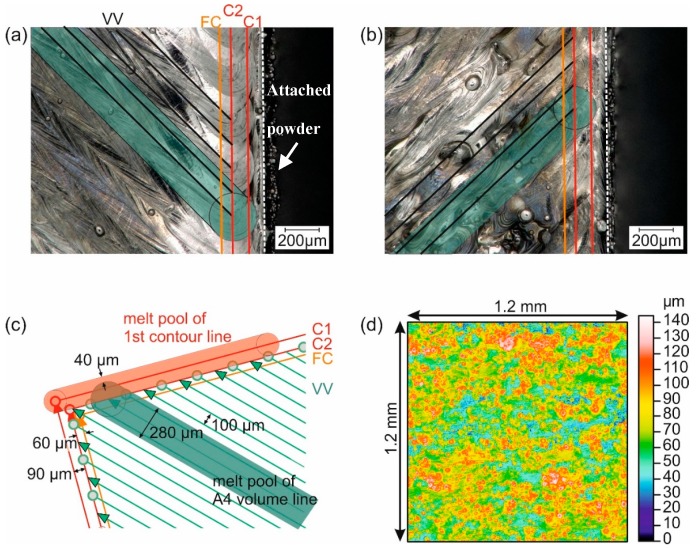
(**a**) Light optical microscopy image of sample A4 (with scan vectors and melt track width marked (C1, C2: first and second contour line; FC: fill contour; VV volume vector); (**b**) light optical microscopy image of A10 with scan vectors and melt track width marked; (**c**) schematic view of the scan strategy in the wall region showing contour, fill contour, and bulk laser vectors; (**d**) roughness map of the lateral surface of sample A4 (*E*_v_ = 116.7 J/mm^3^).

The structure of the near-surface outer layers of the SLM samples is critical for the interpretation of the residual stress condition in these regions (Figure 5a,b). Firstly, partially molten powder particles attached to the sample walls, as well as the outer contour lines, need to be accounted for, simply because they render the identification of the surface position difficult. As for the attached powder particles, they represent an inhomogeneous layer of thickness between 20 μm and 50 μm. Moreover, during fabrication, the two outer contour lines (C1 and C2) are initially melted by the laser, followed by the fill contour (FC), and then finally the bulk region (VV), see Figure 5c. The outer and fill contour scan strategies are similar for all samples: the first and second contour lines, C1 and C2, are separated by 90 μm, while the fill contour FC is further offset by 60 μm. All three lines are laser-treated with a power of 100 W and a laser scan velocity of 525 mm/s, resulting in a melt track width of ~180 μm. The bulk scan lines are defined to end at the central second contour line.

Depending on the energy density of the bulk SLM scanning parameters, the bulk melt tracks display large widths (i.e., ~280 μm for A4, ~250 μm for A10); thus, their ends extend far beyond the second and into the first contour line region (Figure 5a,b). For sample A4, a small contour wall element of <40 μm width remains discernible, whereas on sample A10, a larger remainder is observed. However, it must be kept in mind that the roughness measurements showed values of *S*_a_ and *S*_q_ around 20 μm (see Table 3).

**Figure 6:**
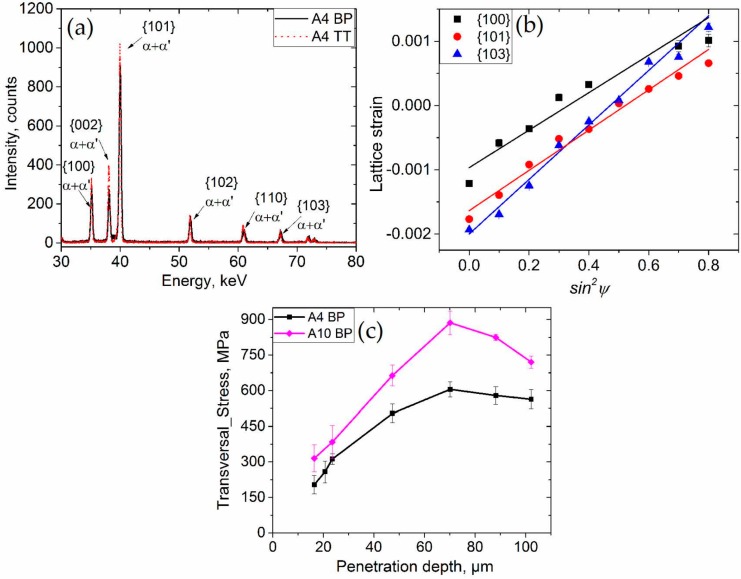
(**a**) Diffractograms for samples with the baseplate (BP) and after thermal treatment (TT); (**b**) example of sin^2^ ψ data evaluation for A4 BP (*L* = 4, *T* = 0.5); (**c**) transversal component of residual stress profile as a function of depth for samples with BP.

The synchrotron diffraction measurements provide diffraction patterns with several reflections of the α + α’ hexagonal close packed Ti lattices (Figure 6a,b). Although in the literature the assessment of phase stresses in the phase has been shown to be possible [35], only the 200 reflection of the body-centred cubic phase is (weakly) discernible after annealing at 650 °C for 3 h (not shown), evidencing that the residual stress is driven by the hexagonal phases. The lattice strain versus sin^2^ ψ diagram (Figure 6b) shows a linear tendency, i.e., little effect of the texture, thereby also confirming the absence of shear stresses.

For an assessment of the residual stress state, it is necessary that the penetration depth during the diffraction experiment exceeds the roughness and contour regions (Figure 6); it should reach at least 100 μm into the sample bulk to probe the direct effects of the SLM parameters on the stress state beyond the contour region. This would also ensure that the gauge volume is fully immersed in the sample. The typical (representative for all scanned positions) stress profile for the transversal direction as a function of depth is presented in Figure 6c. Both samples show a linear increase of stresses with depth until maxima of ~600 MPa and 875 MPa at around a 70 μm depth are reached for A4 (*E*_v_ = 116.7 J/mm^3^) and A10 (*E*_v_ = 53 J/mm^3^), respectively. Then, the stress in the A4 condition remains fairly constant, while it decreases down to ~700 MPa for the A10 condition at ~100 μm from the surface. The A10 condition presents higher stresses in comparison with A4, for the same penetration.

**Figure 7:**
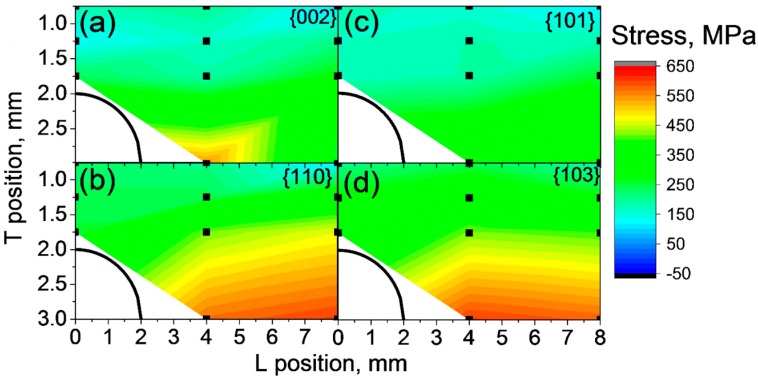
Transversal residual stress maps for different crystallograpic planes for condition A4 BP (**a**) {002}; (**b**) {110}; (**c**) {101} and (**d**) {103}. The black line in the left bottom corner indicates the opening of the bridge with the pillar to the right. The average error is ±30 MPa.

The transversal stress components measured in the SLM bridges in an “as-built” state on the baseplate (BP) in the T direction exhibit consistently significant tensile residual stresses, with higher values occurring towards the pillar bottom (Figure 7). The average error of residual stress maps is around ±30 MPa. The basal plane {002} (Figure 7a) and the pyramidal plane {101} (Figure 7c) present a similar distribution of tensile stresses as the prismatic {110} (Figure 7b) and the pyramidal {103} planes (Figure 7d). The stress increase towards the pillar is much more pronounced for the {110} and {103} planes, reaching stresses around 650 MPa. The crystallographic plane {103} is only weakly influenced by intergranular stresses, i.e., less plastic anisotropy [36], providing an accurate assessment of the macroscopic residual stresses. Thus, {103} was chosen for further analysis. 

**Figure 8:**
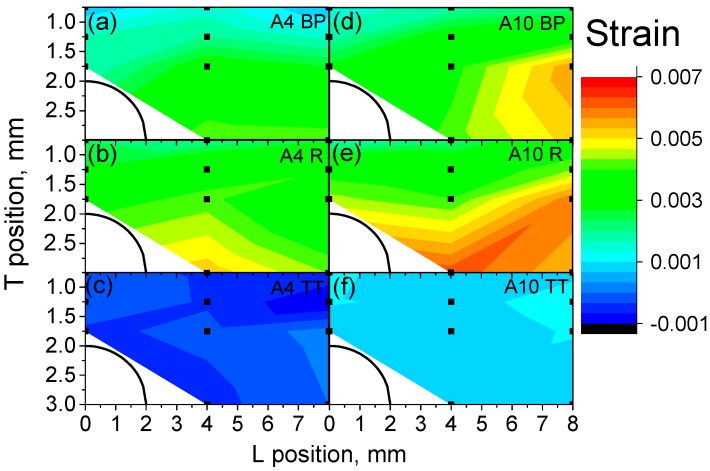
Lattice strain maps of crystallographic plane {103} in the T direction (building direction) for process conditions A4 (**a**; BP) with baseplate, (**b**; R) released, (**c**; TT) after thermal treatment and A10 (**d**; BP) with baseplate, (**e**; R) released, (**f**; TT) after thermal treatment. The average error is ±4 × 10^−4^.

Lattice strain maps are plotted in Figure 8, in order to compare them with the distortion measurements in Figure 4. The average error of the lattice strain is about ±4 × 10^−4^ (relative error below 10%). The lattice strains in the T direction for A10 BP-with lowest energy density-(Figure 8d) are larger than those of the corresponding positions of A4 BP (Figure 8a). A4 BP shows a positive strain gradient from the top surface (ε_T_ ~ 0.0015) to the middle of the sample (ε_T_ ~ 0.005; Figure 8a). After releasing from the baseplate (R), the strains in sample A4 R (Figure 8b) remain similar to those of A4 BP. In contrast, the comparison of sample A10 BP to A10 R shows a broadening of the high-strain region of the pillar after release (Figure 8d,e). The heat treated samples (650 °C for 3 h) show decreased strain values to almost zero, and no further gradients are observed in both A4 and A10 (Figure 8c,f). This implies an efficient relief of residual stresses from the BP condition.

**Figure 9:**
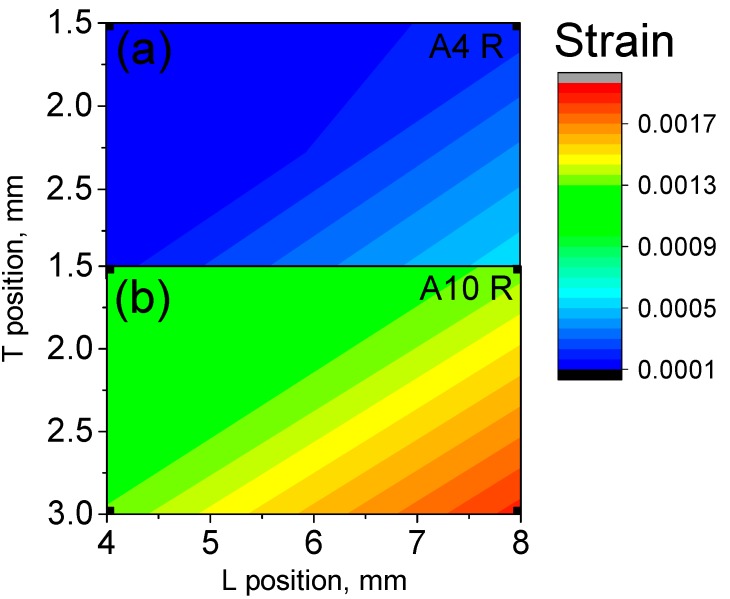
Longitudinal components of residual strains for crystallographic plane {103} for the pillar sections of samples (**a**) A4 R and (**b**) A10 R. The average error is about ±3 × 10^−4^.

In the case of samples A4 R and A10 R, it was also possible to characterize the longitudinal component of the lattice strains (Figure 9). The average error of the longitudinal lattice strain was around ±3 × 10^−4^. It must be noted that, due to the elongation of the gauge volume, the middle-top part above the arc was not measured and only the results for the pillar region are reported (Figure 2 and Figure 3b).

The longitudinal lattice strain components in both samples A4 R and A 10 R show tensile strains with increasing values going from the arc to the pillar. Interestingly, the longitudinal strains are almost an order of magnitude lower than the transversal ones: the maximum strains found for A4 are ε_L_ = 0.0007 (versus transversal strain ε_T_ = 0.005), and for A10 ε_L_ = 0.002 (versus transversal strain ε_T_ = 0.006). A4 R presents significantly lower longitudinal strains than A10 R, as found for the transversal component.

## 4. Discussion

The residual stress state in the SLM Ti-6Al-4V samples shows a strong dependence on: (i) the geometry of the part; (ii) the fabrication parameters (i.e., process energy density *E*_V_); (iii) the heat treatment; and (iv) the removal from the base plate. The lattice strains for the conditions A4 BP (*E*_v_ = 116.7 J/mm^3^) and A10 BP (*E*_v_ = 53 J/mm^3^) consistently show high tensile values on their front surface. These high tensile strains in the subsurface region can lead to crack formation on the surface during mechanical loading.

The effect of the baseplate is manifested by the measurable distortion of samples after releasing from the baseplate (A4 R and A10 R). In fact, the only difference between the stress states in the bridges before and after cutting is the presence of the baseplate. This means ${\overset{=}{\sigma }}_{BP}+{\overset{=}{\sigma }}_{S}={\overset{=}{\sigma }}_{R}$ (tensorial relation), where ${\overset{=}{\sigma }}_{BP}$ is the stress in the bridge before release, $\;{\overset{=}{\sigma }}_{S}$ is the stress in the bridge caused by the presence of the baseplate (before cutting), and ${\overset{=}{\sigma }}_{R}$ is the stress in the bridge after cutting. The contribution of the baseplate to the bridge stress ${\overset{=}{\sigma }}_{S}$ can be calculated knowing the distortion of the bridges after release from the baseplate, i.e., it must hold ${\overset{=}{\sigma }}_{S}=C{\overset{=}{\varepsilon }}_{D}$, where ***C*** is the elastic tensor of the bridge material. Indeed, the residual strain state after cutting, ${\overset{=}{\varepsilon }}_{R},$ must be the sum of the residual strain before cutting ${\overset{=}{\varepsilon }}_{BP}$ and the distortion strain ${\overset{=}{\varepsilon }}_{D}$ (i.e., $\;{\overset{=}{\varepsilon }}_{D}={\overset{=}{\varepsilon }}_{R}-{\overset{=}{\varepsilon }}_{BP}$). In other words, converting the released bridge back to its original form (i.e., applying $-{\overset{=}{\varepsilon }}_{D}$) would restore the original strain field:

(1) $${\overset{=}{\varepsilon }}_{BP}=-{\overset{=}{\varepsilon }}_{D}+{\overset{=}{\varepsilon }}_{R}$$

**Figure 10:**
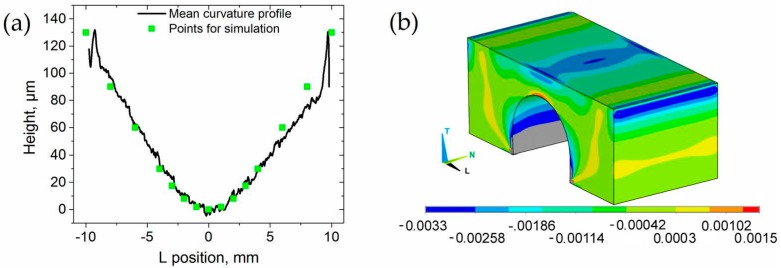
(**a**) Mean curvature profile of A10 specimen with marked data points used for a FE-simulation; (**b**) transversal elastic strain $\varepsilon_{T}$ resulting from the simulation in 3D (initial state: deformed top surface; end state: flattened top surface).

The information of distortion from confocal microscopy measurements on the top surface can be used to calculate the whole strain distribution on the frontal surface of the samples using the finite element method (FEM). Thus, the mean curvature profile (Figure 10a) was implemented via keypoints in the software ANSYS, creating an initial deformed geometry. During the simulation, the top surface was flattened using position dependent displacements in the T-direction (Figure 2a). A linear elastic isotropic material (*E*^103^ = 120.6 GPa, υ^103^ =0.31) was chosen, for which the mesh consisted of 70080 hexahedral elements (SOLID186). Figure 10b represents the resulting transversal elastic strain ($\varepsilon_{TD}$) in 3D for sample A10 R, necessary to counterbalance the measured distortion. In our discussion above, it will hold: $\varepsilon_{TD}=-\varepsilon_{D}$.

**Figure 11:**
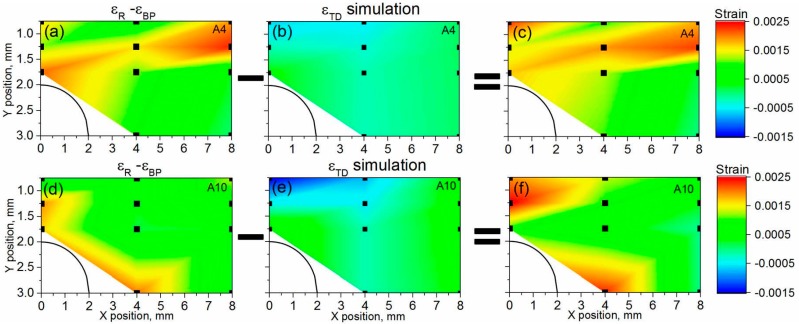
Strain maps for sample A4: (**a**) experimental free distortion component; (**b**) simulated free distortion component; (**c**) the difference between experimental and simulated free distortion; for sample A10; (**d**) experimental free distortion component; (**e**) simulated free distortion component; (**f**) the difference between experimental and simulated free distortion. The average error for experimental data is around ±4 × 10^−4^.

The comparison between strains counterbalancing distortion ($\varepsilon_{TD}$, obtained from the simulations) and the diffraction experimental results ($\varepsilon_{R}-\varepsilon_{BP}$), is depicted in Figure 11. From the simulation, compression strains are present on the top-centre of the bridge, with a tensile counterpart in the inner part of the arc (Figure 11b,e). The distortion-related strain for A10 R (Figure 11e) is larger than for A4 R, and A10 R shows a more pronounced strain gradient above the arc of the bridge. 

The strain difference ($\varepsilon_{R}-\varepsilon_{BP}$, Figure 11a,d) calculated by means of diffraction data (Figure 8) shows higher values near the bridge arc. Figure 11c,f depicts the difference between diffraction-based (Figure 11a,d) and distortion-based strains (Figure 11b,e). This difference should be zero, if we take into account Equation (1). However, the diffraction strain difference (with and without baseplate) does not match the distortion-related strain calculated by FEM. The significant difference could be explained by the model assumption of a simply linear elastic and isotropic material behaviour. Therefore, the diffraction results can also play the role of the benchmark for the model.

The main mechanism leading to the residual stresses in AM parts is the rapid solidification and the heat coming from new layer deposition. Therefore, laser energy, scanning speeds, and laser patterns have a strong influence on the solidification and heat distribution conditions. The slower the scanning speed of the SLM laser, the larger the volume affected by the introduced energy. In turn, the thermal gradients in the specimen decrease. A reduction of the cooling rate due to slower laser scanning has been reported in [37], as well as a smaller thermal gradient and decreasing deflection angles (i.e., distortion obtained by the bridge curvature method [16]) in geometrically identical bridge-shaped samples. This should imply a decrease of residual stresses. Our present study confirms that for a high energy density (samples A4), lower tensile sub-surface residual stresses are found compared to a low energy density (samples A10). In a previous study, the SLM processing window was determined based on the minimization of bulk defects [28]. The samples with the lowest volume fraction of pores coincides with that with lower residual stresses (A4) obtained in this study. The dependence of the deformation angles on energy density (Figure 4) would indicate that lowering velocities further will not substantially decrease the stress state, but lead to increased material defects in the form of keyhole pores [28,38,39]. The analysis of the trend of sample distortion (Figure 4), including the additional SLM parameters A1 (*E*_v_ = 291.7 J/mm^3^) and A3 (*E*_v_ = 145.8 J/mm^3^), indicates that the residual stress reaches a plateau-like minimum when the laser scan velocities decrease to 500 mm/s (A4) and below (A3).

In the investigated regions of both samples, only tensile residual stresses were found in all as-built conditions; however, it should be mentioned that only subsurface regions were investigated. The origin of high tensile residual stress in the subsurface region is reported in [40]. In that study, the authors explain this effect through layer-by-layer rapid solidification and cooling phenomena. Previous (destructive) characterization of residual stresses in SLM Ti-6Al-4V parts by means of the contour method [22] showed that the largest principal stresses are those along the building direction. Furthermore, high tensile stresses near the yield stress appeared towards the surfaces/edges of the specimens. These tensile stresses on the edge of the samples were balanced by compressive stresses in the bulk. Similar residual stress profiles (tensile towards the surfaces and compression in the bulk) have been extensively reported for conventional welds [41] and also for laser direct metal deposited Ni-base superalloy parts (e.g., [42]). 

The thermal gradients for lower SLM scan speeds are more favourable for achieving lower initial residual stresses, although this limits productivity by increasing the building time. Lower stress states are critical when building complex-shaped components. Near the baseplate, a sufficiently strong attachment by means of direct building on the base or by strong supports can prevent deformations, i.e., allows achieving accurate near-end shape geometries while preventing parts from bending, and potentially disturbing the SLM build job. Moreover, with larger build heights and with higher geometrical complexity, support structures in-between are needed. Those are minimized or ideally completely suppressed in order to reduce the difficult and costly post-processing steps for their removal. Hence, lower initial stresses would facilitate production of complex shapes such as turbo-engine impellers with fine, three-dimensionally curved blades and poorly accessible undercuts.

## 5. Conclusions

Synchrotron energy dispersive measurements, in combination with angle distortion characterization, have proved a suitable approach for assessing residual stress fields in SLM Ti-6Al-4V, supporting the process and post-process development. Tensile stresses built up at the near-surface region during SLM for all studied conditions. After heat treatment, those stresses were fully relieved.

We found that the processing window minimizing defects also allows the reduction of initial (tensile) residual stresses. This implies smaller component distortion during SLM processing and could lead to more cost-effective post-treatment processes.

Further investigations are underway to study the role of the contour lines (and their variation/elimination) and the effects of surface-post treatments for control in the development of surface residual stresses on SLM parts. 

## Figures and Tables

**Table 1:** SLM scanning parameters employed for the bridge specimen, and resulting porosity fraction. Error in the determination of porosity lies about 0.1 vol %.

Sample	Laser Power, *P* (W)	Hatch, *h* (mm)	Velocity, *v* (mm/s)	Focus, *f* (mm)	Energy Density, *E*_v_ * (J/mm^3^)	Porosity (vol %) [28]
A1	175	0.1	200	0	291.7	3.3
A3	175	0.1	400	0	145.8	0.6
A4	175	0.1	500	0	116.7	0.1
A10	175	0.1	1100	0	53.0	0.7

* *E*_v_ = $\frac{P}{v\cdot h\cdot x}$, *x* = powder layer thickness = 30 μm.

**Table 2:** Diffraction elastic constants of α-titanium calculated with the Eshelby/Kröner model. The single crystal elastic constants were taken from [34].

{*hkl*}	−s_1_ (MPa^−1^ × 10^−6^)	½ s_2_ (MPa^−1^ × 10^−6^)
100	2.979	12.026
002	2.321	10.112
101	2.899	11.799
102	2.720	11.284
110	2.979	12.026
103	2.579	10.870

**Table 3:** Roughness measurements on the bridge specimens A1, A3, A4, and A10 (side surface): *S*_a_, *S*_q_.

Roughness	A1	A3	A4	A10
*S*_a_ (μm)	20.1	17.6	18.2	17.5
*S*_q_ (μm)	23.9	21.4	21.8	20.8
